# Lymphocytes Infiltration and Expression of PD-1 and PD-L1 in Colorectal Cancer Between HIV-Infected and Non-HIV-Infected Patients: A Propensity Score Matched Cohort Study

**DOI:** 10.3389/fonc.2022.827596

**Published:** 2022-03-02

**Authors:** Ye Cao, Qian Wu, Shixian Lian, Li Deng

**Affiliations:** Department of General Surgery, The Shanghai Public Health Clinical Center, Fudan University, Shanghai, China

**Keywords:** HIV, colorectal cancer, tumor-infiltrating lymphocytes, PD-1, PD-L1, multiplex immuno-fluorescent analysis

## Abstract

**Background:**

Tumor-infiltrating lymphocytes (TILs) and expression of programmed cell death 1 (PD-1)/programmed death ligand-1 (PD-L1) are crucial for antitumor immunity. However, the status remains undetermined in HIV-infected colorectal cancer (CRC), limiting the use of immunotherapy in HIV-infected CRC patients.

**Methods:**

We examined 27 HIV-infected patients and 120 non-HIV-infected patients with CRC from 2015-2020 at Shanghai Public Health Clinical Center. After matching the propensity score, 13 paired patients in the two groups were also compared. The expression of PD-1/PD-L1 as well as tumor-infiltrating CD4, CD8, and CD56 immune cells was examined using multiplex immunofluorescent analysis. The cell density for positive staining was calculated (cells/mm^2^) and compared between HIV-infected and non-HIV-infected groups. In addition, the co-expression of PD-1 on immune cells and PD-L1 on tumor cells was compared in these two groups.

**Results:**

The mean densities of tumor-infiltrating CD4, CD8, CD56 immune cells were 620.2, 261.2, and 0.2 cells/mm^2^, respectively, in HIV-infected colorectal tumors compared with 698.6, 243, and 14 cells/mm^2^ in non-HIV-infected tumors. PD-1 expression was 227 cells/mm^2^ in HIV-infected tumors and 365.2 cells/mm^2^ in non-HIV-infected tumors. Besides, PD-L1 expression was 108.5 cells/mm^2^ in HIV-infected tumors and 126.8 cells/mm^2^ in non-HIV-infected tumors, and no significant difference was found between the two groups. Similarly, there were no significant differences in the expression of PD-1 on TILs and PD-L1 on tumor cells.

**Conclusion:**

HIV-infected CRC patients had similar tumor-infiltrating lymphocytes (CD4 and CD8 T cells) compared to non-HIV-infected controls and substantially similar PD-1 expression on TILs and PD-L1 expression on tumors. These results support the inclusion of HIV-infected CRC patients in future immunotherapy trials.

## Introduction

Since the 1980s, the human immunodeficiency virus (HIV) has spread rapidly throughout the world, infecting totally more than 70 million people and 1.7 million new infections annually (https://www.unaids.org). In the past, most HIV-infected patients died from acquired immunodeficiency syndrome (AIDS)-related diseases, including AIDS-defining malignancies (ADMs) such as Kaposi’s sarcoma, non-Hodgkin’s lymphoma, and cervical cancer in women (1). This was changed by introducing combination antiretroviral therapy (cART), which enabled HIV-infected patients to live relatively normal life (2). However, it was found that the incidence of non-AIDS-defining malignancies (NADMs) increased, including primary cancers in liver, lung, colorectal, breast, prostate, and Hodgkin’s lymphoma (3–7).

Colorectal cancer (CRC) is the most common tumor and has a worse prognosis in HIV-infected patients compared to HIV non-infected patients (7, 8). Traditionally known risk factors include obesity, excessive consumption of red meat, family history, and hereditary polyposis. Additionally, it is believed that HIV-induced immunodeficiency might increase CRC incidence (9). Nevertheless, numerous studies have revealed that CRC incidence does not differ between HIV-infected and non-infected individuals (10, 11), and there is no increase in CRC among HIV-infected individuals with low CD4 cell numbers (12). Additionally, Coghill reported that CRC incidence among HIV-infected patients in North America was only 51%-69% of the normal population (13). These different clinical findings imply that more studies are required to reveal the relationship between HIV-related immunodeficiency and CRC development (14).

CRC patients can be categorized into deficient mismatch repair proteins (dMMR) or proficient mismatch repair proteins (pMMR) groups. Compared to pMMR tumors, dMMR caused a distinct biological and clinical phenotype in CRC, including significantly increased tumor mutation burden, expression of programmed cell death 1 (PD-1), and its corresponding programmed death ligand-1 (PD-L1), and tumor-infiltrating lymphocytes (TILs) (15). High persistent disease control rates have been reported in patients with dMMR metastatic CRC treated with immune checkpoint inhibitors (ICIs) (16, 17). Despite the detection of lower TILs and insufficient ICIs efficacy in metastatic CRC patients with pMMR, several clinical trials still investigate other combination immunotherapy approaches (18). Unfortunately, CRC patients with HIV were excluded from all clinical trials examining immunotherapy, which were considered by HIV-related immunodeficiency within tumor tissues (19). Since studies have revealed no difference between the expression of TILs and PD-L1 in HIV-infected and non-HIV-infected lung cancer (20, 21), some cases of ICIs treatment for lung cancer with HIV have been reported (19). Therefore, understanding TILs and PD-1/PD-L1 expression in HIV-infected colorectal cancer is essential for developing immunotherapy for CRC patients with HIV. In the present study, PD-1/PD-L1 expression and immune cell infiltration in CRC tissues were analyzed, and correlations with clinical characteristics were examined in HIV-infected and non-HIV-infected patients with CRC.

## Methods

### Patients and Samples Collection

We collected and retrospectively analyzed clinical data of CRC patients undergoing surgery between January 2015 and January 2020 at Shanghai Public Health Clinical Center (SHPHC). This study recruited 147 patients diagnosed with colorectal adenocarcinoma, which were divided into HIV-infected (n=27) and non-HIV-infected (n=120) groups according to their history or HIV antibody test results. The following characteristics were included: age, gender, body mass index (BMI), disease history (hypertension, diabetes, and presence of intestinal obstruction on admission), laboratory tests on admission (tumor markers and plasma lipids), tumor location, pathological stage (using the AJCC 8th edition), dMMR status (using immunohistochemical results of four MMR proteins) and surgical resection with/without tumor residuals. Formalin-fixed paraffin-embedded tumor tissue blocks were obtained from the Department of Pathology of SHPHC, and 5-μm-thick serial sections were generated for subsequent multiplexed immunofluorescence analysis. These studies were conducted according to the Declaration of Helsinki and approved by the Shanghai Public Health Clinical Center.

### Multiplex Immunofluorescence and Image Analysis

Tissue multiplex immunofluorescence staining was performed using Opal 7color IHC staining kit (Akoya Biosciences). Briefly, tissue sections of 5-μm thick formalin-fixed, paraffin-embedded (FFPE) were baked for 2 h at 60°C before staining. Slides were rehydrated with a series of graded ethanol to deionized water. Antigen retrieval was performed at pH 6 for 20 min at 95°C. Subsequently, slides were serially stained with primary antibodies, such as CD4 (1:400, ab133616, Abcam), CD8 (1:200, ab21344, Abcam), CD56 (1:200, ab75813, Abcam), Pan-CK (1:500, ab27988, Abcam), PD-1 (1:200, ab52587, Abcam), and PD-L1 (1:100, ab213524, Abcam), with incubation time for each primary antibody of 60 min. DAPI was used to visualize the nuclei. Subsequently, anti-rabbit/mouse Polymeric Horseradish Peroxidase was applied as a secondary label with an incubation time of 10 min. Antibody signal was visualized by their corresponding Opal Fluorophore by incubating the slides for 10 min. Slides were mounted with anti-fade mounting medium (P36965, Life Technologies) and stored at 4°C. Image acquisition was performed using Vectra Polaris multispectral imaging platform. The entire slide image was scanned, and the pathologist chose representative 8-12 regions of interest at 200× resolution as multispectral images. Image analysis was performed using InForm 2.4.8 Image Analysis Software (22). Algorithms were designed to detect positively stained CD4, CD8, CD56, Pan-CK, PD-1, or PD-L1 cells and estimate the density (positive cells/mm^2^).

### Statistical Analysis

We utilized propensity score matching (PSM) to achieve covariates balance between the two groups and reduce confounding factors that may potentially affect post-analysis. The propensity score, defined as the probability that a CRC patient with HIV-infected versus uninfected on clinicopathological characteristics listed above, was estimated by logistic regression. Then, PSM was performed using 1:1 nearest neighbor matching with a maximum caliper of 0.05 (23). Differences in characteristics between the two groups were examined. Categorical variables were reported as integers and proportions, and continuous variables were reported as medians, means (standard deviations), and interquartile ranges. We used Wilcoxon rank-sum test for continuous variables and chi-square or Fisher’s exact test for classified variables. A two-tailed P < 0.05 was considered statistically significant. All statistical analyses were determined using R software (version 3.6.3, http://www.r-project.org). The R package ‘MatchIt’ was used for PSM.

## Results

### Patient Characteristics

Clinicopathological characteristics prior to propensity score matching showed significant differences between HIV-infected and non-HIV-infected groups (**Table S1**). A higher proportion of males was found in HIV-infected group (81.5% *vs*. 57.5%). The mean BMI of HIV-infected group was lower compared to non-HIV-infected group (21 *vs*. 23.5). The history of hypertension and diabetes was less common in HIV-infected group versus non-HIV-infected group. Preoperative tumor marker testing demonstrated a slightly higher level in HIV-infected group, which could be due to more advanced stages in HIV group (stage IV: 22.2%) than non-HIV-infected group (stage IV: 5.8%). Therefore, the ratio of complete tumor resection (R0) was 81.5% in HIV-infected group and less than 94.2% in non-HIV-infected group. The characteristics of age, tumor location, and dMMR status revealed no considerable differences between the two groups. After propensity score matching, 13 patient pairs were obtained. All baseline characteristics were identical between the two groups, and the differences described above were eliminated (**Table S1**). Finally, five pairs of patients were randomly selected for multiplexed immunofluorescence analysis. All characteristics of the ten paired patients are displayed in **Table 1**, and no difference was detected. Pre- and post-PSM in this study, the mean peripheral blood CD4 cell count of patients in HIV-infected group was approximately 270 cells/µL, which was considered a well-controlled level (>200 cells/µL). The peripheral blood CD8 cell count remained high.

**Table 1:** Patient Characteristics.

Characteristics	HIV (n = 5)	non-HIV (n = 5)	All (n = 10)	Pvalue
**Gender n (%)**				
male	2 (40.0%)	4 (80.0%)	6 (60.0%)	
female	3 (60.0%)	1 (20.0%)	4 (40.0%)	0.52
**Age**				
Mean (SD)	60 (11.7)	55 (7.9)	57.5 (9.8)	
Median [MIN, MAX]	62 [48,77]	54 [44,64]	57.5 [44,77]	0.69
**BMI**				
Mean (SD)	19.3 (2.4)	21.6 (4.7)	20.5 (3.7)	
Median [MIN, MAX]	18.4 [16.8,23]	19.5 [17.1,29.1]	19.4 [16.8,29.1]	0.42
**Intestinal obstruction** **on admission n (%)**				
no	5 (100.0%)	5 (100.0%)	10 (100.0%)	
yes	0 (0.0%)	0 (0.0%)	0 (0.0%)	1.00
**Hyperlipidemia n (%)**				
no	5 (100.0%)	4 (80.0%)	9 (90.0%)	
yes	0 (0.0%)	1 (20.0%)	1 (10.0%)	1.00
**CEA** ^#^				
Mean (SD)	1.8 (1)	4.9 (5.7)	3.4 (4.2)	
Median [MIN, MAX]	1.3 [0.8,3.3]	1.9 [1.1,14.6]	1.7 [0.8,14.6]	0.42
**CA125** ^#^				
Mean (SD)	10.2 (3.6)	19.2 (24.4)	14.7 (17.1)	
Median [MIN, MAX]	10.2 [6.1,15.5]	7.8 [6.5,62.6]	9 [6.1,62.6]	1.00
**CA199** ^#^				
Mean (SD)	12.8 (11.9)	9.2 (6.1)	11 (9.1)	
Median [MIN, MAX]	6.4 [2.6,30.5]	9.9 [1.3,17.4]	8.1 [1.3,30.5]	0.84
**CD4 count** ^#^				
Median [MIN, MAX]	293 [276,552]	–	–	
**CD8 count** ^#^				
Median [MIN, MAX]	469 [360,912]	–	–	
**Tumor location n (%)**				
left	3 (60.0%)	3 (60.0%)	6 (60.0%)	
right	2 (40.0%)	2 (40.0%)	4 (40.0%)	1.00
**T-stage n (%)**				
II	2 (40.0%)	1 (20.0%)	3 (30.0%)	
III	2 (40.0%)	3 (60.0%)	5 (50.0%)	
IV	1 (20.0%)	1 (20.0%)	2 (20.0%)	1.00
**N-stage n (%)**				
0	4 (80.0%)	4 (80.0%)	8 (80.0%)	
I	1 (20.0%)	1 (20.0%)	2 (20.0%)	1.00
**M-stage n (%)**				
0	5 (100.0%)	5 (100.0%)	10 (100.0%)	
I	0 (0.0%)	0 (0.0%)	0 (0.0%)	1.00
**TNM-stage n (%)**				
I	2 (40.0%)	0 (0.0%)	2 (20.0%)	
II	2 (40.0%)	4 (80.0%)	6 (60.0%)	
III	1 (20.0%)	1 (20.0%)	2 (20.0%)	0.68
**dMMR status n (%)**				
no	4 (80.0%)	5 (100.0%)	9 (90.0%)	
yes	1 (20.0%)	0 (0.0%)	1 (10.0%)	1.00
				

^#^means Reference Value Range: CA125: 0-35 U/ml; CA199: 0-37 U/ml; CEA: 0-5 ng/ml;

CD4 count: 410-1590 cell/ul; CD8 count: 190-1140 cell/ul.

### TILs and PD-1/PD-L1 Showed No Difference Between HIV and Non-HIV Groups

**Figure 1:**
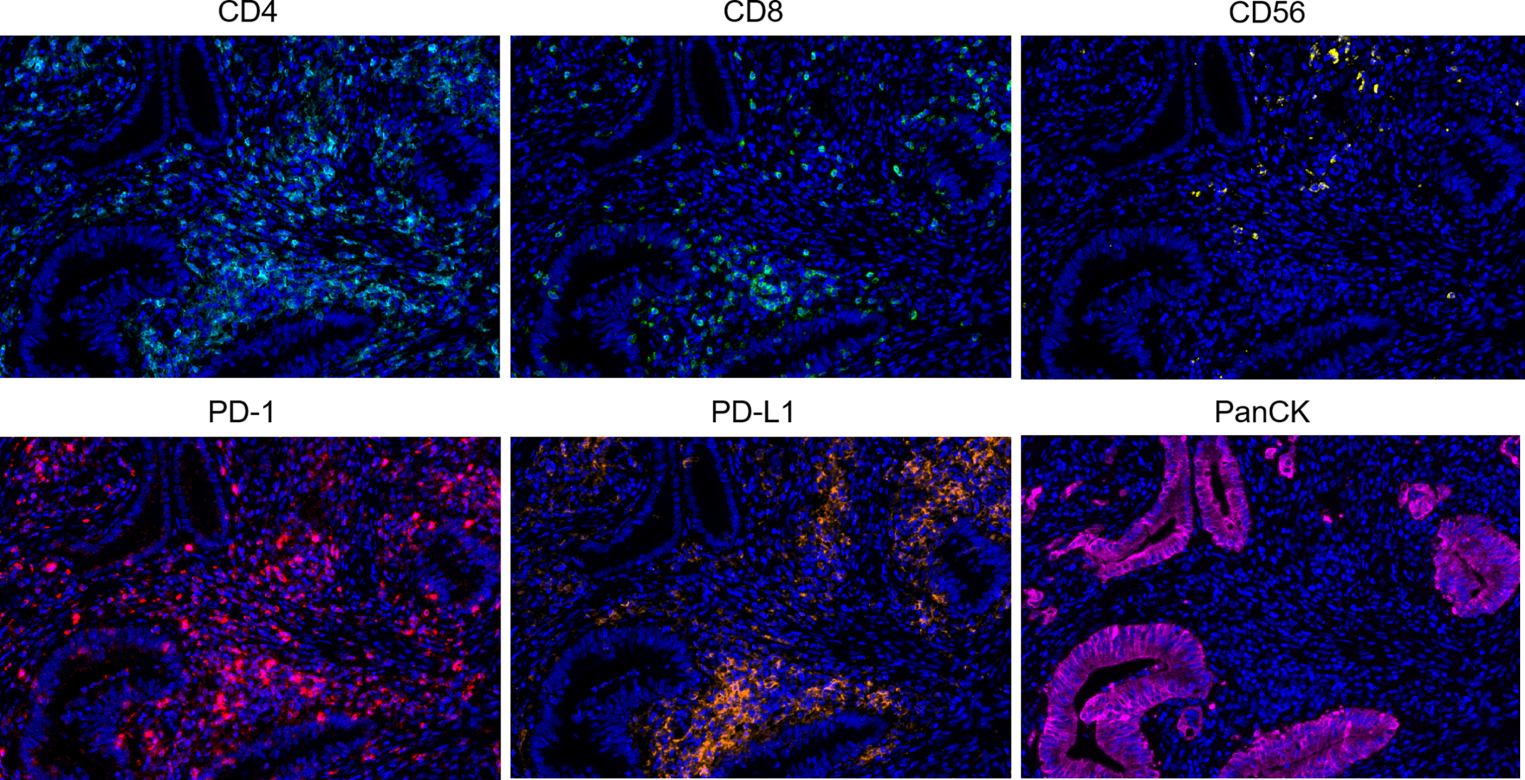
Representative multiplex immunofluorescence images of positive staining in colorectal cancer tissue sections. Color scheme - Cyan: CD4, Green: CD8, Yellow: CD56, Red: PD-1, Orange: PD-L1, Magenta: PanCK, Blue: DAPI.

**Figure 2:**
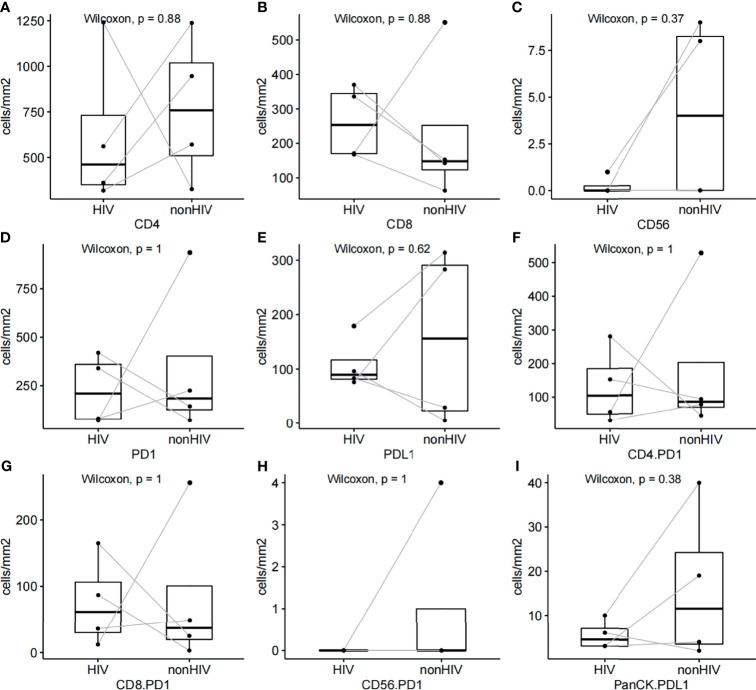
**(A–E)** Pairing test for relative CD4, CD8, CD56, PD-1, and PD-L1 cell densities between HIV-infected and non-HIV-infected CRC samples. **(F–H)** Pairing test of PD-1 positive CD4, CD8, CD56 cell densities between HIV-infected and non-HIV-infected CRC samples. **(I)** Pairing test of PD-L1 positive tumor cell densities between HIV-infected and non-HIV-infected CRC samples.

**Figure 3:**
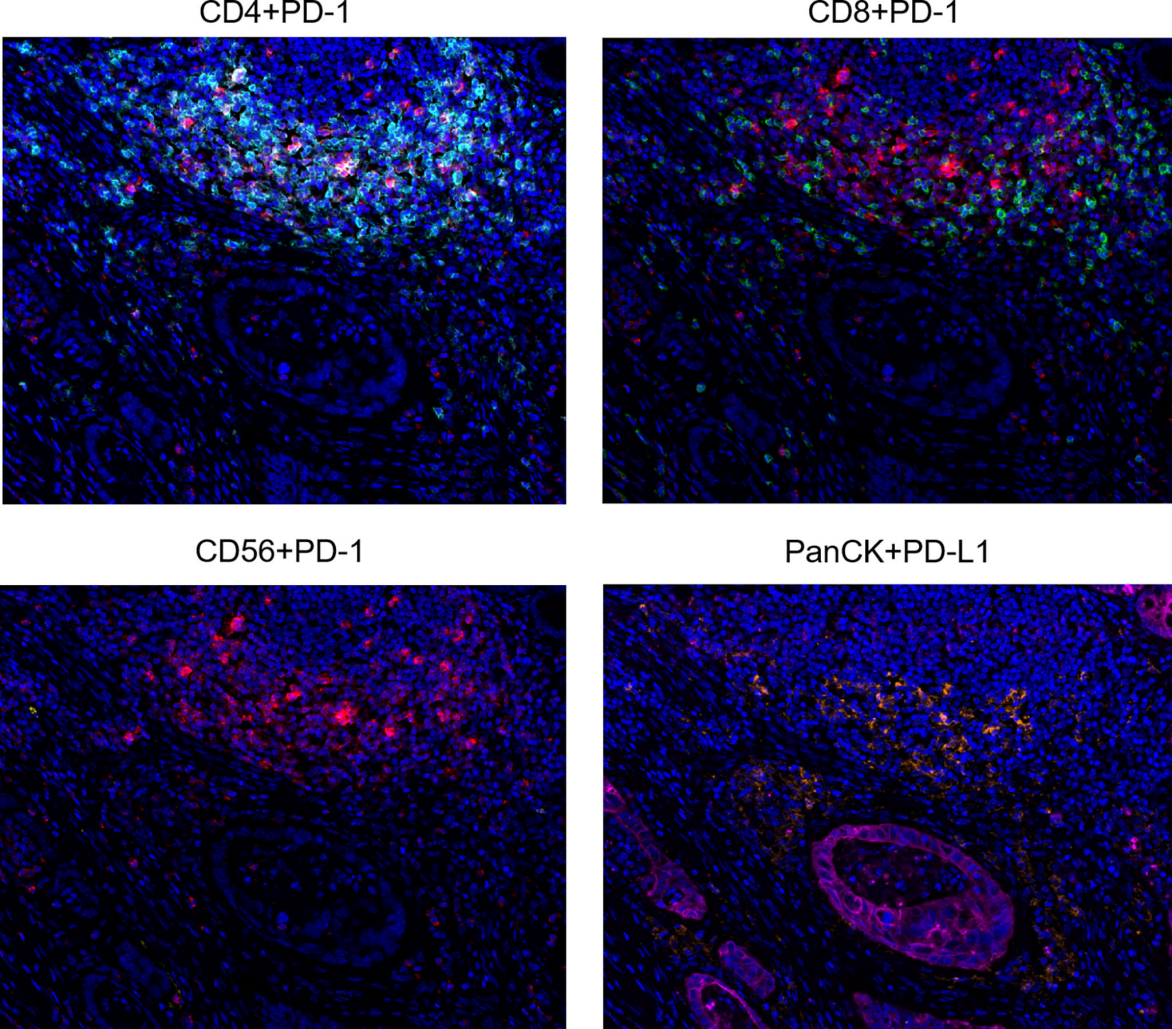
Representative multiplex immunofluorescence images of co-expression of PD-1 on CD4, CD8, CD56 cells, and PD-L1 on tumor cells. Color scheme - Cyan: CD4, Green: CD8, Yellow: CD56, Red: PD-1, Orange: PD-L1, Magenta: PanCK, Blue: DAPI.

Multiplex immunofluorescence was used to enable serial fluorescent labeling of multiple proteins within a single tissue slide, as described above (**Figure 1**). Tumor cells were distinguished by the presence of Pan-CK staining. Using Vectra Polaris imaging platform to quantify cell densities within whole tumor sections (one patient’s tissue section failed to collect data due to quality issues), we determined the average intratumor densities of PD-L1, PD-1, CD4, CD8, and CD56 positive cells (**Table 2**). The density of CD4 cells was 620.2 cells/mm^2^ in HIV-infected group compared to 698.6 cells/mm^2^ in non-HIV-infected group. The density of CD8 cells was 261.2 cells/mm^2^ in HIV-infected group compared to 243 cells/mm^2^ in non-HIV-infected group. The density of CD56 cells was 0.2 cells/mm^2^ in HIV-infected group versus 14.4 cells/mm^2^ in non-HIV-infected group. The density of PD-1 positive cells was 227 cells/mm^2^ in HIV-infected group versus 365.2 cells/mm^2^ in non-HIV-infected group. The density of PD-L1 positive cells was 108.5 cells/mm^2^ in HIV-infected group compared to 126.8 cells/mm^2^ in non-HIV-infected group. No significant differences were found. Furthermore, in the pairing test, intratumor CD56 cells density was lower (P = 0.37) in HIV-infected group compared to non-HIV-infected group (**Figure 2C**); nevertheless, the number of CD56 cells was very low in both groups. However, CD4, CD8, PD-1, and PD-L1 positive cells revealed no significant differences between the two groups (**Figures 2A, B, D, E**). The co-expression of PD-1 positive tumor-infiltrating lymphocytes and PD-L1 positive tumor cells is also demonstrated in **Figure 3** and **Table 2**. The density of PD-1 positive CD4 cells was 130 cells/mm^2^ in HIV-infected group compared to 178.8 cells/mm^2^ in non-HIV-infected group. The density of PD-1 positive CD8 cells was 75 cells/mm^2^ in HIV-infected group compared to 77.6 cells/mm^2^ in non-HIV-infected group. PD-1 positive CD56 cells were very rare in both groups. We also detected the number of PD-L1 positive tumor cells and found that PD-L1 was rarely expressed on tumors in these selected cases. No difference was found in the matched-pair test for PD-1 positive TILs (**Figures 2F–H**). The correlation of different immunocytes of HIV-infected group was shown in **Figure S1**. Only CD4+ cell density correlated strongly with the CD4/CD8 ratio in tumors and in peripheral blood.

**Table 2:** Summary of Cell Density Measurements.

	HIV (n = 4)	non HIV (n = 5)	All (n = 9)	P value
**CD4 (cells/mm** ^2^)				
Mean (SD)	620.2 (427.2)	698.6 (383.9)	663.8 (379.2)	
Median [MIN, MAX]	461 [318,1241]	571 [326,1238]	561 [318,1241]	0.730
**CD8 (cells/mm** ^2^)				
Mean (SD)	261.2 (106.9)	243 (193.1)	251.1 (151.7)	
Median [MIN, MAX]	253.5 [168,370]	153 [63,551]	171 [63,551]	0.413
**CD56 (cells/mm** ^2^)				
Mean (SD)	0.2 (0.5)	14.4 (23.1)	8.1 (18)	
Median [MIN, MAX]	0 [0,1]	8 [0,55]	0 [0,55]	0.227
**PanCK (cells/mm** ^2^)				
Mean (SD)	4256.5 (1399.7)	5256.4 (2283.9)	4812 (1902.7)	
Median [MIN, MAX]	4550.5 [2528,5397]	4944 [2371,8609]	4944 [2371,8609]	0.730
**PD1 (cells/mm** ^2^)				
Mean (SD)	227 (179.7)	365.2 (350.2)	303.8 (280.6)	
Median [MIN, MAX]	209.5 [69,420]	225 [70,937]	225 [69,937]	0.556
**PDL1 (cells/mm** ^2^)				
Mean (SD)	108.5 (47.7)	126.8 (157.5)	118.7 (115.5)	
Median [MIN, MAX]	89.5 [76,179]	29 [3,314]	83 [3,314]	0.730
**CD4+PD-1 (cells/mm^2^)**				
Mean (SD)	130 (113.7)	178.8 (200)	157.1 (159.8)	
Median [MIN, MAX]	104.5 [30,281]	93 [44,530]	93 [30,530]	0.905
**CD8+PD-1 (cells/mm^2^)**				
Mean (SD)	75 (67.7)	77.6 (101.8)	76.4 (83.1)	
Median [MIN, MAX]	61.5 [12,165]	49 [3,256]	49 [3,256]	0.905
**CD56+PD-1 (cells/mm^2^)**				
Mean (SD)	0 (0)	5 (9.1)	2.8 (7)	
Median [MIN, MAX]	0 [0,0]	0 [0,21]	0 [0,21]	0.240
**PanCK+PD-L1** **(cells/mm^2^)**				
Mean (SD)	5.5 (3.3)	13.2 (16.7)	9.8 (12.6)	
Median [MIN, MAX]	4.5 [3,10]	4 [1,40]	4 [1,40]	1.000
**Total (cells/mm** ^2^)				
Mean (SD)	11133.5 (1092.9)	11776.8 (1238.4)	11490.9 (1153.1)	
Median [MIN, MAX]	11610 [9508,11806]	12119 [10343,13484]	11742 [9508,13484]	0.413

## Discussion

With the development of effective cART, the lifespan of people living with HIV is greatly prolonged. The number of HIV-infected people aged 65 years or older is expected to increase once in the next decade. The proportion of NADMs that accounted for about 20% of all tumors in the pre-cART era has reached 70%. In the next ten years, one of NADMs will be more common than all ADMs combined (24). Nevertheless, cART does not completely remove HIV from the infected patient. If cART is stopped, HIV rapidly rebounds and continues to destroy the immune system. Regarding efficacy and tolerance of cancer drugs when combined with cART, the standard cancer treatment remains lower for people living with HIV (19).

The neo-antigen-targeting antitumor immune response is sometimes potent enough to eradicate the tumor by infiltrating the immune cells. Regarding colorectal cancer, tumors with high infiltrate of CD8 and T helper 1 (CD4) type cells had a better prognosis than those with a low infiltrate (15, 25). However, immune checkpoint proteins suppress the immunological attack against tumor cells, such as PD-1. Blocking the interaction between PD-1 on immune effector cells and PD-L1 on tumor cells has been demonstrated to reverse the suppression of antitumor immunity and has revealed significant antitumor effects in different malignancies, including advanced colorectal cancer (CRC) (17). It was found that CRC patients had a poorer prognosis within HIV-infected than non-infected patients (7). From the standpoint of antitumor immunity, HIV infection-induced immunosuppression is likely to be associated with poor CRC prognosis. However, conclusive evidence for this assumption has not yet been obtained. This study compared immune- and cancer-related antigens in CRC tissue specimens of patients with and without HIV infection. We did not observe a significant difference in the number of positive cells including CD4, CD8, and CD56 and the number of cells expressing PD-1 and PD-L1 between propensity score-matched cohorts. Therefore, it was hypothesized that HIV infection does not affect lymphocytic infiltration within the tumor. In contrast, the reason for the poor prognosis of colorectal cancer with HIV is uncertain, possibly due to the initial diagnosis at an advanced stage (**Table S1**) and inadequate treatment (8).

HIV infection is associated with the progressive loss of T cells, and functional impairment of HIV-specific CD8 and CD4 T cells ultimately leads to the inability of the host immune system to maintain control of HIV, accelerating disease progression (26). One reason for T cell exhaustion is increased PD-1 expression on the surface of CD4 and CD8 T cells and inhibitory ligand PD-L1 on cells of a myeloid lineage (27). Continuous viral antigen stimulation persists even in patients receiving antiretroviral therapy, resulting in sustained activation of HIV-specific CD8 T cells (28). Tumor-specific CD8 T cells can become PD-1 positive cells by the influence of continuously activated HIV-specific CD8 T cells (21). The observations support that PD-L1/PD-1 interactions play a key role in regulating the immune environment in HIV infection and may induce an immune environment favorable for tumor development. However, these assumptions remain vague and lack evidences. In the present study, we compare the co-expression of PD-1 on CD4, CD8, and CD56 tumor-infiltrating cells, and PD-L1 on tumor cells between HIV-infected and non-HIV-infected groups. Without immunosuppression of T cells in peripheral blood, we did not identify upregulated expression of PD-1 in tumor-infiltrating lymphocytes and PD-L1 in tumor cells. Peripheral blood CD4/CD8 ratio is used as an indicator to monitor the severity of HIV infection. This value was closely associated with CD4 tumor-infiltrating cells, but was not observed to affect PD-1 and PD-L1 expression in tumors (**Figure S1**). In a study of non-small cell lung cancer, it was demonstrated that tumors in HIV patients probably had a greater sensitivity towards immunotherapy (29). Consequently, further studies are required to elucidate the underlying mechanisms, especially in colorectal cancer.

HIV infection remains an exclusion criterion for cancer clinical trials of ICIs. Prospective data on the safety and activity of these novel anticancer agents in people living with HIV (PLHIV) are unavailable. In case reports, treatment with ICIs in PLHIV revealed similar impacts and side effects as in non-infected individuals, and no enhancement of HIV replication was observed (19). However, these studies have mainly focused on non-small cell lung cancer and solid tumor, for which data on colorectal cancer treatment are lacking. We hope that by describing the immune cell infiltration and expression of PD-1/PD-L1 in HIV colorectal cancer patients, we can provide theoretical evidence for future development of ICIs treatment.

Approximately 15% of CRC patients have poorly differentiated dMMR tumors and have histologic heterogeneity and increased tumor-infiltrating lymphocytes compared to pMMR tumors (15, 30). However, the microenvironment of pMMR tumors still presents activation on prime antigen-specific CD4 and CD8 T cells of cancer immunity (31, 32). In this study, only pMMR tumors were selected for immunofluorescence analysis, and the accumulation of CD4 and CD8 T cells within tumors was observed. We did not detect a decrease in T-cell infiltration in HIV group, which may provide a theoretical basis for subsequent development of enhanced therapy with effector T-cell (33, 34). We also plan to continue recruiting dMMR-CRC cases in a follow-up study to investigate whether there are differences in TILs between groups. Another limitation of the present study is the small number of patients selected for multiplexed immunofluorescence analysis, which may influence the outcomes. By utilizing propensity scores and pair testing, patient backgrounds were widely equalized to minimize the influence.

## Conclusion

HIV-infected colorectal cancer (CRC) patients had similar tumor-infiltrating lymphocytes (CD4 and CD8 T cells) compared to HIV-uninfected controls and significantly similar PD-1 expression on TILs and tumor PD-L1 expression. No significant immunosuppression was detected in the tumor microenvironment with well-controlled HIV infection. Our findings support the inclusion of HIV-infected CRC patients in future clinical trials evaluating the efficacy of immunotherapy or immune checkpoint inhibitors directed against these tumor-specific T cells or molecules.

## Data Availability Statement

The original contributions presented in the study are included in the article/**Supplementary Material**. Further inquiries can be directed to the corresponding authors.

## Ethics Statement

The studies involving human participants were reviewed and approved by Shanghai Public Health Clinical Center. The patients/participants provided their written informed consent to participate in this study.

## Author Contributions

LD conceived the study. LD and SL co-wrote the paper. YC and QW collected all data and undertook the search. All authors contributed to the article and approved the submitted version.

## Funding

The present study was supported by the clinical research program of Shanghai Public Health Clinical Center (KY-GW-2021-20).

## Conflict of Interest

The authors declare that the research was conducted in the absence of any commercial or financial relationships that could be construed as a potential conflict of interest.

## Publisher’s Note

All claims expressed in this article are solely those of the authors and do not necessarily represent those of their affiliated organizations, or those of the publisher, the editors and the reviewers. Any product that may be evaluated in this article, or claim that may be made by its manufacturer, is not guaranteed or endorsed by the publisher.

## Supplementary Material

Supplementary Figure 1 The correlation of different immunocytes in HIV-infected CRC samples. The circles and colors in the upper right part indicate the correlation between each two types, and the values in the lower left part indicate the p-value after Pearson’s test.
