# Supplementary figures and images for: Lymphocytes Infiltration and Expression of PD-1 and PD-L1 in Colorectal Cancer Between HIV-Infected and Non-HIV-Infected Patients: A Propensity Score Matched Cohort Study
Source: Front Oncol. 2022 Mar 2;12:827596. doi: 10.3389/fonc.2022.827596 (PMC8924460; doi:10.3389/fonc.2022.827596)

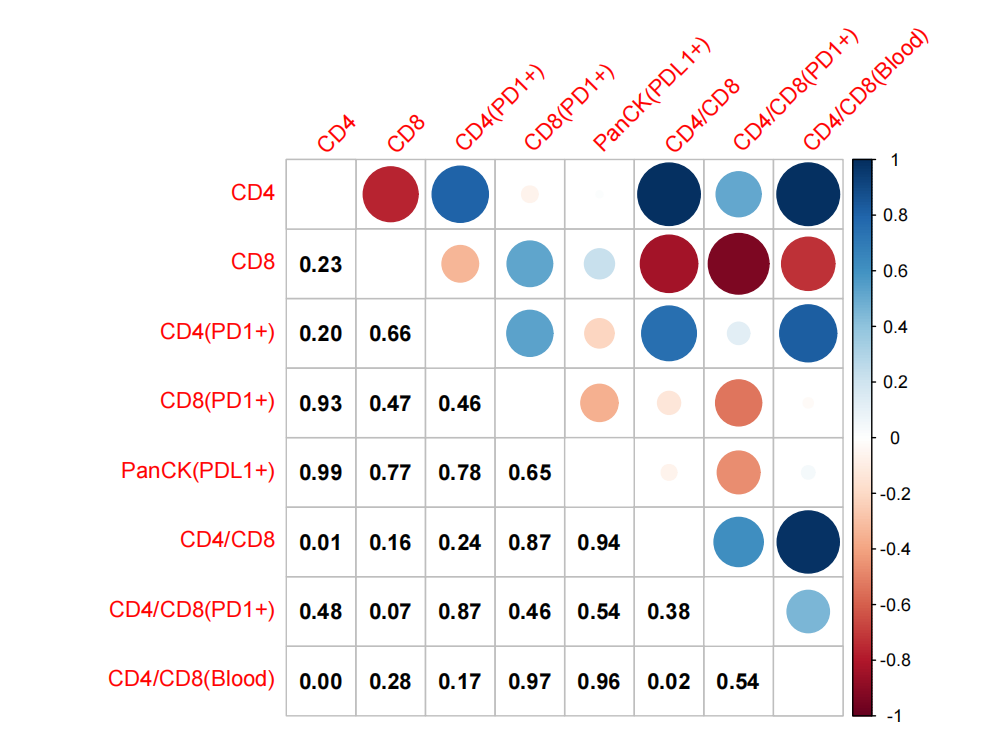

Supplement: Supplementary Figure 1 — The correlation of different immunocytes in HIV-infected CRC samples. The circles and colors in the upper right part indicate the correlation between each two types, and the values in the lower left part indicate the p-value after Pearson’s test. [file Image_1.tif]
